# Microbe-Host Interactions: Structure and Role of Gram-Negative Bacterial Porins

**DOI:** 10.2174/138920312804871120

**Published:** 2012-12

**Authors:** Stefania Galdiero, Annarita Falanga, Marco Cantisani, Rossella Tarallo, Maria Elena Della Pepa, Virginia D’Oriano, Massimiliano Galdiero

**Affiliations:** 1 Department of Biological Sciences, Division of Biostructures, University of Naples “Federico II” and Istituto di Biostrutture e Bioimmagini, CNR, Via Mezzocannone 16, 80134, Naples, Italy; 2 Department of Experimental Medicine, Division of Microbiology - II University of Naples, Via De Crecchio 7, 80138, Naples, Italy

**Keywords:** Porin, bacteria, immunobiology, signaling pathways, structure.

## Abstract

Gram negative bacteria have evolved many mechanisms of attaching to and invading host epithelial and immune cells. In particular, many outer membrane proteins (OMPs) are involved in this initial interaction between the pathogen and their host. The outer membrane (OM) of Gram-negative bacteria performs the crucial role of providing an extra layer of protection to the organism without compromising the exchange of material required for sustaining life. The OM, therefore, represents a sophisticated macromolecular assembly, whose complexity has yet to be fully elucidated. This review will summarize the structural information available for porins, a class of OMP, and highlight their role in bacterial pathogenesis and their potential as therapeutic targets.

The functional role of porins in microbe-host interactions during various bacterial infections has emerged only during the last few decades, and their interaction with a variety of host tissues for adhesion to and invasion of the cell and for evasion of host-defense mechanisms have placed bacterial porins at the forefront of research in bacterial pathogenesis. This review will discuss the role that porins play in activating immunological responses, in inducing signaling pathways and their influence on antibiotic resistance mechanisms that involve modifications of the properties of the OM lipid barrier.

## INTRODUCTION

Infectious diseases are major threats to human health worldwide, and tremendous effort is continuously devoted to the understanding of various infectious agents and their mechanisms of virulence. The field of bacterial pathogenesis is a rapidly evolving and expanding one. 

The mammalian immune system has in place a line of defense specialized in recognizing and eradicating invading pathogens; however, sometimes the pathogen evades these mechanisms and establishes diseases in its host. The treatment of bacterial infections with antibiotics is one of the key concepts of human medicine. Therapies like antibiotics and vaccination support the immune system in its fight against pathogenic microbes. After several decades of continued success of antibiotic therapy, we are now facing a worrying prospect: the accelerated evolution of antibiotic resistance of important human pathogens, which presents a global health problem with a strong social and economic impact. This bacterial adaptation to antibiotic use is directly involved in the current increase of morbidity and mortality caused by infection diseases. Over time, resistance to antibiotics has developed due to the intense selective pressure the antibiotics place on bacteria. Furthermore, while a number of vaccines have been successful, far too many infectious diseases still do not have efficacious vaccines. An urgent need for new therapeutics exists and understanding the complex relationship among the host and invading pathogens will provide important insight for the rational design of therapeutics.

Gram negative bacteria comprise a major proportion of drug resistant pathogens and display a complex envelope with an outer (OM) and an inner (IM) membrane delimiting a periplasmic space. This cellular organization results in the presence of various protein channels involved in the transport, uptake or efflux, of a large variety of compounds, nutrients or toxic molecules (sugars, drugs, small peptides, chemicals). 

The outer membrane is the first line of defense for Gram-negative bacteria against toxic compounds. This barrier is impermeable to large, charged molecules. Influx is largely controlled by porins, which are water-filled open channels that span the outer membrane and allow the passive penetration of hydrophilic molecules. Bacterial pathogenicity is largely dependent on its surface structures. Among the components of the bacterial outer membrane, outer membrane proteins (OMPs), such as the porins, play a fundamental role in pathogenicity and in protection and represent useful targets for therapeutic development [1-3].

Porins are involved in the exchange of nutrients over the outer membrane of Gram-negative bacteria but are also involved in pathogenesis. The present review will focus on the description of the structure and the role of Gram-negative bacterial porins in the bacteria-host interactions. 

## DESCRIPTION OF BACTERIAL ENVELOPES

**Fig. (1):**
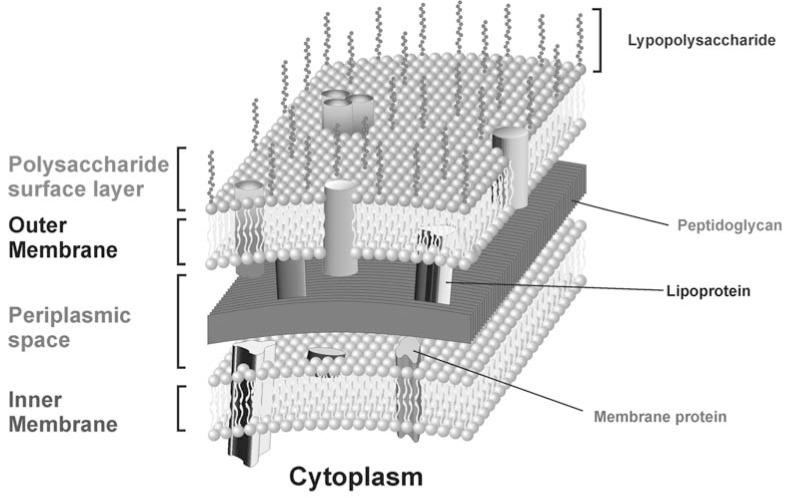
Schematic representation of the inner and outer bacterial membrane.

Bacteria in order to face unpredictable and often hostile environments have evolved a sophisticated and complex cell envelope that protects them while allowing selective passage of nutrients from the outside and waste products from the inside. In particular, Gram-negative bacteria can be divided into several subcellular compartments [4]. There are three principal layers: the outer membrane (OM), the peptidoglycan cell wall, and the inner membrane (IM). The two membrane layers delimit two aqueous compartments called the cytoplasm and the periplasm. Present throughout these compartments are proteins with diverse and important biological functions (Fig. **1**). Some of these proteins are membrane-embedded and allow the transfer of molecules between compartments. Others are soluble enzymes involved in metabolic reactions. Much work has been devoted toward understanding how each of these compartments is formed and maintained. The OM is a characteristic feature of Gram-negative bacteria, and in fact Gram-positive bacteria lack this structure. The OM is a lipid bilayer intercalated with proteins, superficially resembling the plasma membrane; it contains phospholipids confined to its inner leaflet, while the outer leaflet contains glycolipids, mainly lipopolysaccharide (LPS). 

**Fig. (2):**
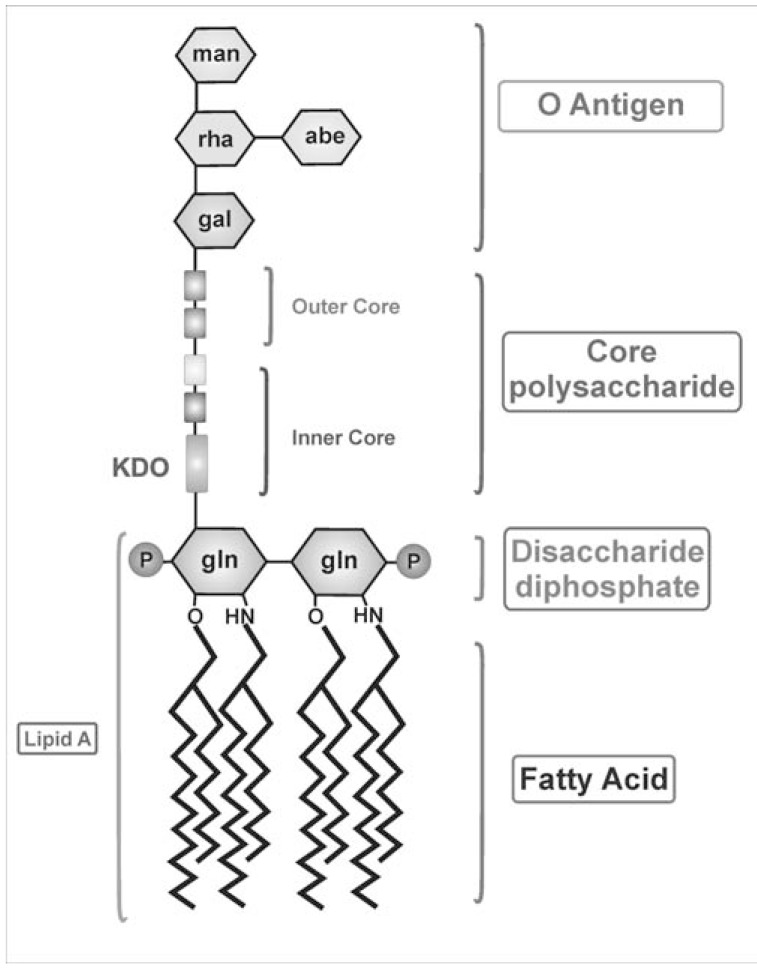
Schematic representation of the structure of lipopolysaccharide (LPS).

The barrier property of the OM can be largely attributed to the presence and asymmetrical distribution of the complex glycolipid LPS. In fact, LPS monomers in the bilayer exhibit a strong lateral interaction with other LPS molecules, these interactions together with the enrichment of fully saturated phospholipids in the inner leaflet of the OM greatly reduce the OM fluidity, which is rigid and gel-like in comparison to the inner membrane. LPS molecules are made of three distinct regions: the lipid A, the core oligosaccharide and the distal O-antigen (Fig. **2**). Lipid A secures LPS in the OM; the core is covalently bound to the lipid A through an acidic sugar, the 3-deoxy-D-manno-oct-2-ulopyranosonic acid (Kdo). The general pattern of the lipid A from diverse Gram-negative bacteria is highly conserved. The core oligosaccharide is very variable among bacterial species; so different species can express uniquely modified types of LPS. The O-antigen, if present, is the most variable part of LPS and shows even a high degree of variability between different strains of the same species.

With few exceptions, the proteins intercalated in the OM can be divided into two classes, proteins that traverse the membrane and assume a β-barrel structure and lipoproteins, anchoring the outer membrane to the underlying peptidoglycan stratum (Fig. **1**). Lipoproteins contain lipid moieties that embed lipoproteins in the inner leaflet of the OM, and are thus not supposed to be transmembrane proteins. The outer membrane proteins (OMPs) of Gram-negative bacteria have been well characterized and many assume a β-barrel conformation. The OMPs serve as a molecular filter for hydrophilic substances, mediate the transport of nutrients and ions across the membrane into the periplasm, some small β-barrels seem to serve primarily as membrane anchors or to promote bacterial adhesion to mammalian cells, or are membrane-bound enzymes such as protease, phospholipase.

The most abundant proteins of the bacterial outer membrane are porins which are essentially trimeric β-barrels forming channels with various grades of selectivity. Porins form passive pores that do not bind their substrates; they generally form water-filled pores, through which relatively small (<600 Da) solutes diffuse, driven by their concentration gradient. For nutrients that are present at low concentrations in the extracellular environment, passive diffusion is no longer efficient and transport occurs via substrate-specific and active transporters [5-9].

## BACTERIAL PORINS: STRUCTURE

**Fig. (3):**
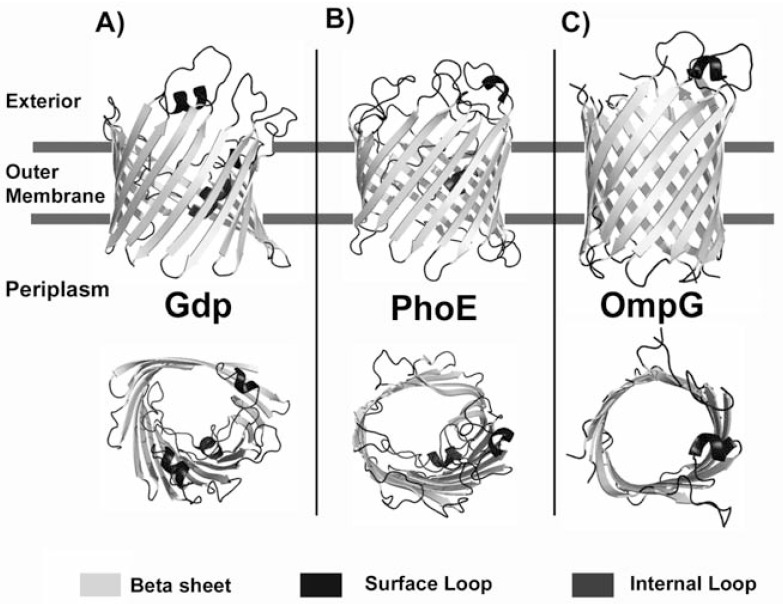
Three dimensional structures of three different porins: Gdp from *Rhodobacter capsulatus*, PhoE and OmpG from *Escherichia coli*. Surface and internal loops are shown in dark grey. The extracellular space is located at the top of the figure and the periplasmic space is at the bottom.

Bacterial porins have been studied in great detail since many years; the first high-resolution X-ray structure was published in 1990/1991 and was the major outer membrane protein of *Rhodobacter capsulatus*. The structure of this trimeric porin showed the archetypical fold of 16 tilted β-strands, all of which are connected by extraplasmic loops and periplasmic turns with the particularly long loop L3 folded inside the barrel (Fig. **3A**) [10,11]. Many additional porin structures have been determined since then with a high similarity in architecture and only smaller variations, mainly in loop topology and surface charges [12-15]. Porins made of 16 strands were classified as general or non-specific porins and form pores allowing the diffusion of hydrophilic molecules, showing no particular substrate specificity, despite some selectivity for either cations or anions; while 18 strands porins were classified as substrate specific porins, both of which are trimeric (Fig. **3B**) [6,7]. This concept has been proved useful for many years and provided a clear structure-based classification into e.g. sugar-specific channels, such as ScrY or LamB with 18 β-strands, or unspecific channels with 16 strands involved in the uptake of small and mostly inorganic molecules (e.g. Omp32, OprP and PhoE) [16-18]. However, during recent years and owing to the discovery of new bacterial cell envelopes and outer membrane protein structures, the group of porins became more diverse and a simple classification seems more difficult. Recently OmpG or CymA (14-stranded monomeric proteins) [19,20] showed a different fold and quaternary structure (Fig. **3C**). In addition, a number of porins previously classified as unspecific were later proved to bind small, typically negatively charged (organic acids or phosphates) ligands, thereby providing a structural and functional rationale for the facilitated small metabolite uptake in bacteria and their specific lifestyle conditions [16,17]. 

**Fig. (4):**
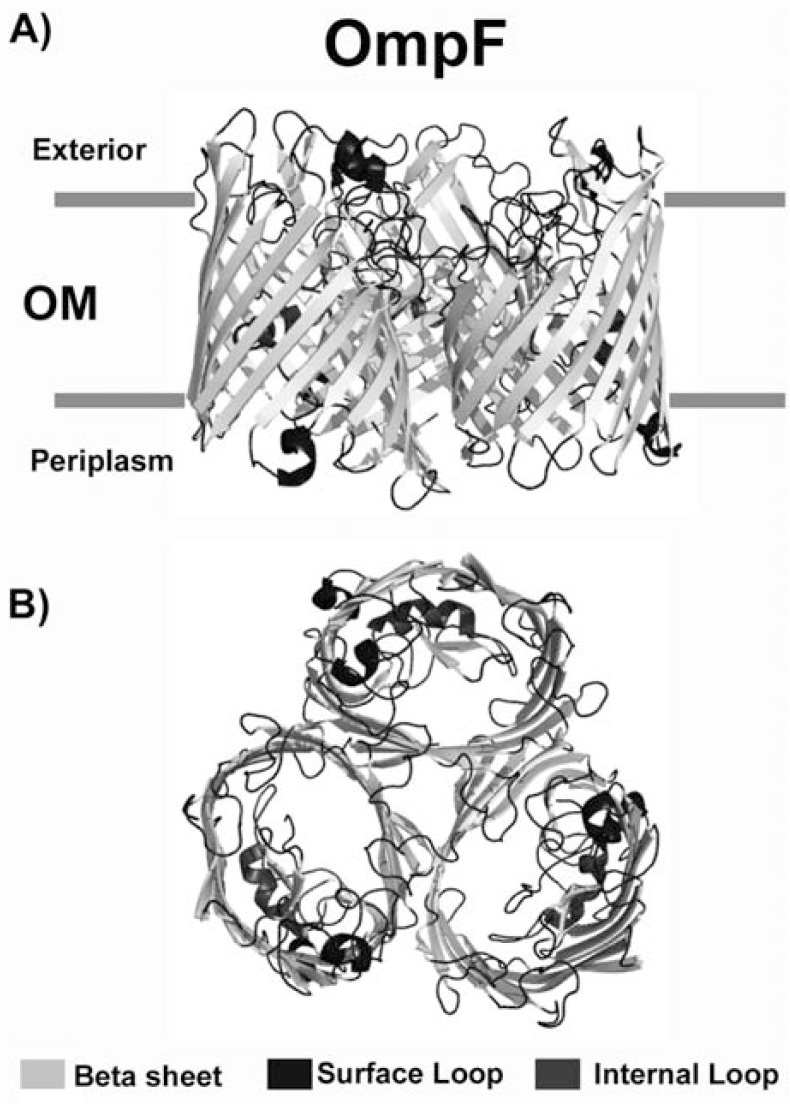
Three-dimensional structure of the porin OmpF from *Escherichia coli*. Surface and internal loops are shown in dark grey. The extracellular space is located at the top of the figure and the periplasmic space is at the bottom. The trimer is shown.

The majority of porins studied so far is typically of oval shape and have an overall dimension of laterally ∼30–35Å and ∼50 Å in height for the monomer. All these proteins share similar properties, such as their high abundance in native membranes, an increased thermal stability and the regular content of β- sheet structures (in the range of 60%), as well as their specific conductance profiles in planar membranes [15]. The general characteristic of their structural architecture is the closure of the barrel by pairing of the first and last β-strand in an antiparallel way. All strands are connected by eight or nine long loops, facing the extracellular side, with seven or eight small turns in the periplasmic space. In all porins, the constriction at the barrel center is formed by an inserted long loop L3, which is not exposed to the cell surface but folds back into the barrel, forming a constriction zone at half the height of the channel and contributing significantly to the permeability of the pore. The type of residues outlining the channel determines the specificity of the pore. All porins form homotrimers in the OM; each subunit produces a channel and the trimer therefore contains three channels (Fig. **4**). For most porins, loops L1, L2 and L4 are important for monomer-monomer interactions within the porin trimer; loop L3 is internal; loops L5, L6 and L7 are superficial; loop L8 folds back into the barrel interior, contributing to the formation of the channel opening at the external side (Fig. **4A**). Peptide sequences corresponding to superficial loops are responsible for most of the biological activity of porins. Another feature is the presence of aromatic girdles with tyrosine and phenylalanine residues located at the outer and inner membrane boundaries. Tyrosine residues are more frequent at the extraplasmic sites, whereas phenylalanine residues are located at the periplasmic side of the highly asymmetric membrane [21]. These aromatic girdles are also present in α-helical membrane proteins and presumably adjust secondary structure elements at the borders of natural membranes [22]. Residues located between these girdles and facing the hydrophobic lipid environment mainly have high Kyte–Doolittle values (e.g. leucine, valine and isoleucine). Additional girdles, often charged, such as observed in Omp32, exist in bacterial porins and may have some implications in a tighter interaction with lipid head groups or with LPS molecules [15]. At the very C-terminus almost all porins have a phenylalanine residue, which is an important prerequisite for proper import and folding in the outer membrane [23,24]. 

The pore diameter ranges from 15 Å for the general porins to 6 Å for the highly selective porins. Larger pores usually contain charged residues at opposite sides that form a local transversal electric field at the pore eyelet. This field constitutes an energy barrier for low-polarity solutes so that the bacterium can exclude unwanted nonpolar molecules such as antibiotics while presenting a spacious eyelet for collecting large polar molecules such as sugars. A systematic study changing the pore properties by point mutations showed a strong correlation between the eyelet cross section and diffusion rate [25]. Charge reversals affect selectivity and voltage gating. Interesting results were obtained with mutations at loop L3, for example the specificity of the sucrose porin was changed toward that of the maltoporin.

The outer membrane contains also porins with specificity for certain substrates. The best known proteins of this class are the sucrose-specific ScrY from *Salmonella typhimurium *[7,26] and the maltooligosaccharide-specific maltoporin LamB from *Escherichia coli *[27]. Both form homotrimers with monomers consisting of 18-stranded antiparallel β-strands, with loop L3 folding back inside the barrel and with hydrophilic surface exposed loops; unlike general porins, their β-barrel contains 18 (rather than 16) strands, and some of the external loops coalesce to form an umbrella that shields the underlying pore and restricts its entrance, transforming it in an elongated pore which may adapt to the shape of maltodextrins. 

Although porin structures typically contain 16 or 18 β-sheets depending on the definition of the protein family [28]. Recently several outer membrane proteins have been classified as porin although their structure is characterized by a number of strands lower than 16 (8, 12 and 14 strands) and are also monomeric. Other barrel proteins with fewer strands have been investigated, most of which have a specific function not related to the diffusion of hydrophilic molecules and are not classified as porins. These proteins are instead designed for the exchange of hydrophobic molecules, such as lipids or hydrophobic substances (e.g. toluene) and are typically closed by loop or cork-domain-like structures [29,30,31]. 

The structure of the 14-stranded porin OmpG from *Escherichia coli* has been determined by X-ray crystallography [20,32]. In E. coli the main porins for sugar uptake are LamB [33] and ScrY [7]; in mutants where LamB is either nonfunctional or deleted, the uptake of sugars is facilitated by OmpG [32]. OmpG has all features of porins: a signal sequence of 21 amino acids at its N-terminus, which is cleaved during export, absence of long hydrophobic stretches, lack of cysteine residues, and a C-terminal phenylalanine, which is important for membrane insertion. OmpG is a monomer and there is no evidence to suggest a physiological oligomer. Owing to a missing loop L3, present in the archetypical porins, the pore opening is considerably large. A long extracellular loop assumes two distinct, well-defined conformations, apparently in response to the pH of the medium. At neutral pH this loop projects into the extracellular medium, leaving the pore wide open, whereas at low pH it folds across the pore channel and blocks it, suggesting a direct role in pH dependent pore-gating.

NanC belongs to the family of small monomeric related porins [34]. NanC folds into a 28 Å high, 12-stranded β-barrel, resembling the β-domain of autotransporter NalP and defining an open pore with an average radius of 3.3 Å. The channel is lined by two strings of basic residues facing each other across the pore, a feature that appears largely conserved within the substrate specific autotransporter family and is likely to facilitate the diffusion of acidic oligosaccharides. Also AlgE from *Pseudomonas aeruginosa* [35] involved in the secretion of newly synthesized alginate across the outer membrane is a monomeric 18-stranded β-barrel. It is characterized by a highly electropositive pore constriction formed by an arginine rich conduit that likely acts as selectivity filter for the negatively charged alginate polymer. Interestingly, the pore constriction is occluded on both sides by extracellular loop L2 and an unusually long periplasmic loop, T8. 

OprP from *Pseudomonas aeruginosa* is involved in the high-affinity acquisition of the concentrations of phosphate that are crucial for *Pseudomonas* growth and proliferation [17]. Each monomer of OprP adopts a 16-stranded antiparallel slightly elliptical β-barrel structure and it forms a prominent trimer in solution, but unlike most of porins, OprP has an extended periplasmic N terminus that is involved in stabilizing the trimer through a ‘tricorn’-like strand exchange. Three prominent elongated loop regions are evident in OprP structure, the extracellular loops L3 and L5 and the periplasmic loop T7. The L3 loop contains α-helices that extend deep into the cavity of the barrel and are responsible for the size and constriction of the pore, as seen in other membrane-spanning β-barrel proteins. Uniquely in OprP, the L5 loop runs along the inner surface of the pore toward the center of the channel, creating an electropositive surface to attract anions. In L5, there are five arginine residues that, together with two arginine residues in sheet B2 create a distinct, evenly spaced seven-step arginine ladder, creating an electropositive slide that propels the phosphate down the inner region of the exoplasmic surface toward the constriction zone. On the extracellular surface of the OprP trimer, each monomer contributes to the formation of a giant funnel. The funnel has three separate electropositive arginine ladders that spiral down toward the point of greatest constriction near the periplasmic face of the transporter.

MspA is a channel protein present in the outer membrane of mycobacteria that has been classified as a porin and is involved in the uptake of small hydrophilic nutrients [36]. The atomic structure of this porin shows a β-structure that differs completely from its counterparts in Gram-negative bacteria. The structure is an octamer containing a central channel of 16 β-strands with a diameter of 40Å; the base of the barrel contains a second short 16-stranded β-barrel that forms a channel constriction, also called a pore eyelet with a diameter of 28 Å. This barrel is similar to that of pore forming toxins such as α-hemolysin [37,38]. 

Among the porin channels are also found proteins such as the P100 protein from *Thermus thermophilus*. Initially this protein was reported to form the S-layer (surface protection layer) of this bacterium [39], later it was shown to form a densely packed structure of porin molecules in native membranes and to be a multi-domain structure made of a N-terminal peptidoglycan binding domain, a long coiled-coil domain and an unusually large porin domain [40,41]. 

## ASSEMBLY AND OLIGOMERIZATION

Studies of bacteriorhodopsin [42] have largely contributed to the comprehension of the assembly and folding processes of α-helical membrane proteins; on the contrary, even though there is a growing understanding of the complex folding process of β-barrel membrane proteins, progress has been much slower. The membrane insertion process is the rate-limiting step, because of the side chain and secondary structure rearrangements required for pore formation. 

Proteins that have to reach the OM must be transported from the ribosome to the inner membrane, where they must be discriminated from inner-membrane proteins, then transported across the inner membrane into the periplasm, carried across the periplasm and finally assembled within the outer membrane in their mature conformations [43]. OMPs are synthesized in the cytoplasm with N-terminal cleavable signal peptides that target them for delivery to the periplasm and thanks to their low hydrophobicity they are presumably exported into the periplasm just like any other periplasmic protein; then, they are refolded into their stable β-barrel conformations and are inserted into the outer membrane. It is not clear whether the refolding process precedes insertion or vice versa, but nascent, monomeric porins can be assembled into the final trimer *in vitro*, in the presence of phospholipids. It has been proposed that the central part of the homotrimer including all N and C termini folds in the periplasm like a water soluble protein so that the membrane facing parts of the β-barrels dangle as 200 residue loops into the solvent. On membrane insertion, these loops can easily meander forming the special β-sheet topology. Studies on OmpA [44-47] provided most of the information on the folding process of outer membrane proteins. The insertion and folding reactions of OMP occur spontaneously without the need of accessory proteins *in vitro*; however, the folding kinetics are relatively slow suggesting that folding *in vivo* might be facilitated by folding catalysts;[24] in particular, membrane insertion and folding of OmpA was most efficient at specific molar ratios of OmpA, Skp and LPS [48]. The water-soluble form of OmpA is unstructured and upon membrane interaction, a membrane-bound intermediate is formed, which is localized at the bilayer interface and is composed of single or paired β-strands with dynamic and metastable hydrogen-bonding contacts. A new intermediate is formed with some of the characteristics of β-barrels which only after an extensive amount of rearrangements assumes the mature structure. 

Its insertion into the outer membrane is mediated by a protein complex that contains the OMP BamA and four associated lipoproteins (BamBCDE) [49-51]. The mechanism by which the Bam complex catalyzes the assembly of OMPs is not known. Bam is a heteropentamer composed of a very highly conserved OM β-barrel (BamA) and four OM lipoproteins (BamBCDE) that dock with BamA. BamA and BamD are indispensable in *E. coli*, and depletion of either leads to rapid accumulation of unassembled OMPs in the periplasm followed by cell death. The periplasmic domain of BamA is essential for its function; it binds the C-terminal signal sequences of β-barrel OMPs, which contain a C-terminal phenylalanine or tyrosine residue. Binding of an OMP causes a conformational change in the C-terminal domain of BAM, which allows the OMP to insert into the outer membrane. Dissociation of the BAM subunits releases the assembled OMPs into the outer membrane, where final conformational changes in the cell-surface-exposed loops may be induced by interactions with the lipopolysaccharides.

Thus, the Bam complex recognizes the C-terminal motif in β-barrel proteins and before its role became clear, it was already detected in porins and autotrasporters. There is a general mechanism for autotransport, where the different types of autotransporters follow the general route for β-barrel protein insertion into the OM. An extended signal peptide in many cases ensures slow processing by the Sec machinery, to gain time for proper OM insertion before the passenger domain is released. Moreover, premature folding in the periplasm is inhibited by the known periplasmic chaperone systems, and also by sequence intrinsic properties of the passenger polypeptides, such as a reduced folding rate, high solubility of the unfolded passenger domains and little to no propensity to aggregate when in the unfolded state. Most probably already during membrane insertion a hairpin structure is formed, and the sequential folding of the passenger domain on the cell surface drives the process to completion.

## ROLE OF BACTERIAL PORINS

During their interaction with the host, porins from several Gram-negative bacteria have diverse biological activities on several eukaryotic cell types, in fact, porins can be considered important inducers of biological activity in host-cell interactions [52]. Several studies have been performed to analyze their immunobiological activities, showing that porins have important effects in several pathogenic mechanisms. Since comprehensive reviews describing the functional behavior of porin channels are available [28], we will mainly focus on three aspects of their role: a) immunological activity of porins, b) porins induced signaling pathways, and c) porins influence on the emergence of antibiotic resistant strains of pathogenic bacteria.

## IMMUNOBIOLOGY OF BACTERIAL PORINS

Although the structural features and function of porins have been well studied, their role in the pathogenesis of various bacterial infections has emerged only during the last decade. Bacterial porins interact with a variety of host tissues for adhesion to and invasion of the cell and for evasion of host-defense mechanisms and are able to elicit innate and acquired immune responses.

*S. typhimurium* porins inhibit phagocytic activity in a dose dependent fashion by activating the adenylate cyclase system [53] and are able to induce the activation of the complement system by acting both on the classic pathway and on the alternative pathway [54]. These porins are clearly endowed with pro-inflammatory activity, in fact, when injected into the paw of male Wistar rats induced dose-dependent edema with long-lasting effects and the induced inflammation is sensitive to both steroid (dexamethasone) and non-steroid (indomethacin) anti-inflammatory drugs. *In vitro* studies carried out on rat resident peritoneal cells showed that porin-induced inflammation may depend on the release of histamine, even though the arachidonic acid metabolites may also participate. In fact, *in vitro* results exclude an increase of 6-keto-prostaglandin and subsequent prostacyclin release, whereas *in vivo* results confirm both the prolonged duration of porin-induced edema and its marked inhibition by indomethacin. Porin-induced inflammation was also observed in decomplemented animals. Further *in vivo* experiments were carried out by intraperitoneal inoculation of 100 µg/ml of porins in guinea pigs [54], therefore, it is unlikely that the activation of the complement system plays a major role in the inflammation induced by porins [54]. Recognition of pathogenic bacteria by host toll-like receptors (TLRs) is the first step in the activation of the inflammatory responses of the innate immune system [55,56]. 

While CD-14, CD-11/18 and Toll-like receptors 2 and 4 appear to be very important LPS signal transducer, porin-specific receptors are still unknown. Therefore, it is possible that porin stimulation is not due to binding to specific receptors, but the consequence of the perturbation of the cell membrane lipoproteic phase, induced during adsorption or porin penetration. CD-14 is a glycosyl-phosphatidyl inositol linked 55 kDa protein present on the surface of monocytes and polymorphonuclear leucocytes, and it functions as the cell surface receptor for LPS and several surface components of Gram-positive bacteria. CD14 is also found as a soluble protein (sCD14) in human serum. CD14 lacks transmembrane and cytokine-binding domains and is not believed to have intrinsic signaling capabilities. TLRs make up a family of evolutionary conserved pattern recognition molecules that are important signal transducers for the induction of mammalian innate immunity responses, including cytokine responses. TLR2 is involved in the recognition of a wide assay of bacterial products, including peptidoglycan, lipopeptides, zymosan and bacterial lipoproteins, whereas TLR4 is activated by LPS. CD14 acts as a broad specificity coreceptor that can enhance cell activation induced by TLR4 or TLR2 agonists. In many cases, porins interact with host immune cells and can be considered as pathogen associated molecular patterns (PAMPS) due to their ability to signal via TLR molecules and other pattern recognition receptors. 

TLR1, TLR2, TLR6, and MD2 have each been suggested to be involved in the recognition of a broad range of OMPs [57-61] . Data from *Haemophilus influenzae* (Hib) porin and from *Neisseria* porin (PorB) indicate that porins from different bacteria may be recognized by TLR-2 [58]. The Hib porin-induced TNF-α and IL-6 production was eliminated in macrophages from TLR2 or MyD88 deficient mice. In contrast, macrophages from LPS hyporesponsive C3H/HeJ mice which are defective in TLR4 function, responded normally to Hib porin. Neisserial porin adjuvant activity was mediated by surface expression of B7-2 and class 2 major histocompatibility complexes on B cells by TLR-2-dependent mechanisms; the presence of the adaptor molecule MyD88 was also required. CD11/18 [58] integrin may also participate in LPS signaling. These families of receptors are heterodimer cell surface glycoproteins composed of a CD11 and a CD18 subunit. The release of TNF-α, IL-6 and IL-8 by THP-1 cells stimulated by porins is independent of CD14, but is partially dependent on CD11/18 integrins.

The basis for the recognition of OMPs by TLRs is difficult to envision because OMPs vary in sequence, structure, diameter, and conductance. However, the analysis of the crystal structures of PorB and the TLR1/2 heterodimer [62] allowed the development of a model for the initial recognition of OMPs by TLR1/2. PorB and other Gram-negative porins have been shown to contain a ring of positively charged residues on the extracellular side of the protein that interacts with the negatively charged lipopolysaccharides to stabilize the porin within the bacterial outer membrane [15,63]. An electrostatic analysis of the TLR1/2 heterodimer revealed that both ectodomains, which mediate recognition, are predominantly negatively charged [64]. The negatively charged surface could be attracted to the ring of positive charges from PorB by nonspecific electrostatic attraction.

Several reports have shown that porins are able to induce signal transmission, activation of nuclear factors, activation of gene promoters and finally release of cytokines. Porins by *Salmonella enterica serovar typhimurium* induce the release of TNF-α, IL-1, IL-6, and TGF by macrophages and IL-4 and IFN-γ by lymphocytes. *Salmonella enterica serovar typhimurium* porins enhance the synthesis and release of IL-6 in U937 cells regulating the transcriptional activity of IL-6 gene by nuclear transduction of NF-κB. The characterization of the human IL-6 promoter revealed a highly conserved control region of 300bp upstream of the transcriptional initiation site that contains the elements necessary for its induction by a variety of stimuli commonly associated with acute inflammatory or proliferative states. In particular, electrophoresis mobility shift assay, as well as promoter deletion and point mutation analysis, revealed the presence of an NF-κB binding element. In U937 cells stimulated by *Salmonella* porins, NF-κB is able to enhance IL-6 gene promoter activity. Activation of this nuclear factor may be responsible for porin induced expression and release of IL-6 [65]. OmpA from *Shigella flexneri* 2a induces the release of proinflammatory cytokines through activation of NF-kB via TLR2 , moreover the induction of IFN-γ expression in CD4+ T cells, through the production of IL-12, in macrophages, demonstrated that OmpA plays a critical role in the development of Th1 adaptive skewed immune response [66].

* Haemophilus influenza* type b (Hib) porin also induces the early release of cytokines in central nervous system cells, amplifying the inflammatory response. Hib porin inserted into the fourth ventricle of the brain elicited the appearance of serum proteins and the development of brain edema. These effects were followed by increase in the number of neutrophils both in cerebrospinal fluid and in the tissue sections around the porin inoculation site. IL-1α, TNF-α and MIP-2 mRNA appeared quickly in the tissue near the inoculation site [67].

As described in this section, porins are deeply involved in inflammation and immune response and several report have shown that mice immunization with porin proteins or DNA immunization with plasmids containing their genes, leads to production of humoral antibodies and also promotes a Th1 cell-mediated immune response. Most functional antibodies raised to non-typable *Haemophilus influenza* (NTHI) are directed to loop L5, which is thought to contain strain-specific and immunodominant epitopes [68], and antibodies to loop L6 of NTHI showed complement-dependent bactericidal activity [69]. Surface exposed loop regions of porins are immunodominant as shown by immunizing mice with whole bacterial cells [70]; synthetic peptides representing epitopes of outer membrane protein F of *Pseudomonas aeruginosa *elicit antibodies reactive with whole cells of heterologous immunotype strains of *Pseudomonas aeruginosa *[71]; major immunogenic epitopes of PorA and FetA of *meningococci* correspond to contiguous peptide sequences located in putative surface-exposed loops of those proteins [72,73]. 

## PORINS INDUCED SIGNALING PATHWAYS

The molecular mechanisms during the interaction of Gram-negative bacteria with macrophages are well understood, but the mechanisms used by porins to activate cells are not well characterized. LPS, porins or other OMPs probably activate cells through similar but not identical mechanisms [2]. The intracellular signaling pathways are complex networks of biochemical events that culminate in specific patterns of nuclear gene expression mediated by transcription factors.

* Salmonella enterica* serovar typhimurium porins induce signal transduction in mouse macrophages [74]. Porin activation of macrophages results in increased inositol triphosphate and intracellular Ca^2+^ mobilization, translocation of protein kinase C (PKC) to the membrane, NO release within the macrophages and increased binding of infected macrophages resulting in macrophage activation and triggering of specific signaling pathways. *Salmonella enterica* serovar typhimurium, *Mannheimia haemolytica*, and *Haemophilus influenzae* (Hib) porins induce tyrosine phosphorylation in THP-1 cells and in C3H/HeJ mouse macrophages [67], with Hib porin being the most powerful stimulator. The pattern of phosphorylation observed following LPS or porin stimulation is essentially similar, but a difference can be observed in the cytoplasmic fraction bands of 50-60 kDa, which are more evident after treatment with LPS, and in the insoluble fraction band of 80kDa and the cytoplasmic fraction band of 250kDa, which are more evident after porin treatment.

Among the most prominent tyrosine-phosphorylated bands in porin-stimulated cells, a number of proteins with a molecular mass that is similar to that of the family of tyrosine/serine/threonine protein kinases were observed. *Salmonella enterica* serovar typhimurium porins stimulation of U937 cells induces tyrosine phosphorylation of ERK1-2, protein kinase A (PKA), PKC and protein-tyrosine kinase (NT-PTKs). In the cells pretreated with tyrphostin, a specific PTK inhibitor, or with H-89, a specific PKA inhibitor, or calphostin C, a specific PKC inhibitor, decrease of the relevant activity was observed [75]. 

Neisserial porins induced protein tyrosine phosphorylation and alter the surface expression of the co-stimulatory molecule B7-2 [76]. Recent evidence suggests that the Raf-1-MEK1/2-MAPK pathways are included among the proteins which are phosphorylated following porin stimulation [77]. The use of some specific inhibitors of phosphorylation pathways such as SB-203580 (p38 inhibitor), PD-098059 (MEK/ERK kinase inhibitor) and forskolin (Raf-1 inhibitor) demonstrated that they modulate in a different way cytokine mRNA expression in cells stimulated with porins. Neisserial porins induce nuclear translocation of the transcription factor NF-κB in B cells and dendritic cells that was maximal by 3 h of stimulation [76]. *Salmonella enterica* serovar typhimurium porins also activated AP-1 and NF-κB in U937 cells involving the Raf-1-MEK1/2-MAPK pathways [77]. Pretreatment with PD-098059 and with SB-203580 markedly affected the activation, indicating that the p38 signaling pathway is mainly involved in AP-1 and NF-κB activation [78]. In fact, PD-098059 is a good and specific inhibitor of MEK-1/2 [79]. In contrast, forskolin pretreatment did not block transcription factor activation by porins, suggesting that a Raf-1-independent pathway may also be involved following porin stimulation. The formation of a different complex represents a further difference between stimulation with LPS and stimulation with porins. This may be added to past observations where mRNA cytokine expression after stimulation with porin begins after 120 min and continues for 5-6 h, while following LPS stimulation begins after 30 min and decreases at 120 min. Forskolin did not block NF-κB translocation after porin stimulation. Raf-1 induces the dissociation of cytoplasmic NF-κB-IκB complexes [80], suggesting that a Raf-1-dependent pathway may be involved in NF-κB activation. In fact, Raf-1 plays a central role in regulating multiple survival pathways in eukaryotic cells and has been also involved in the regulation of the activation of immune effector cells [81]. Moreover, Ras➜Raf-1 mediated axis has been also involved in the triggering of a survival response to antiproliferative and immune regulating cytokines such as interferon alpha in human epithelial cells [82]. However, it is known that PKC triggers the activation of several kinases suggesting that MEK/ERK pathways may also participate in NF-κB activation by enhancing an AP-1-NF-κB cross-coupling mechanism. The porin P2 from *Hib*, like porins from *Salmonella enterica* serovar typhimurium, activates mainly but not exclusively the JNK and p38 pathways. Synthetic peptides, corresponding to the amino acid sequences of variable loop regions facing the cell exterior are responsible for most of the biological activity of porins; in contrast, peptides modeled on internal β-strands were ineffective in inducing phosphorylation of such pathways [83,84]. In particular, loop L7 of porin OMPK36 from *Klebsiella pneumoniae* is involved in the interaction with C1q [85]; loops L5, L6 and L7 of porin P2 from *Haemophilus influenzae* activate JNK and p38 mitogen-activated protein kinase (MAPK) pathways [83] and induce the release of TNF-α and IL-6 [84]. 

**Fig. (5):**
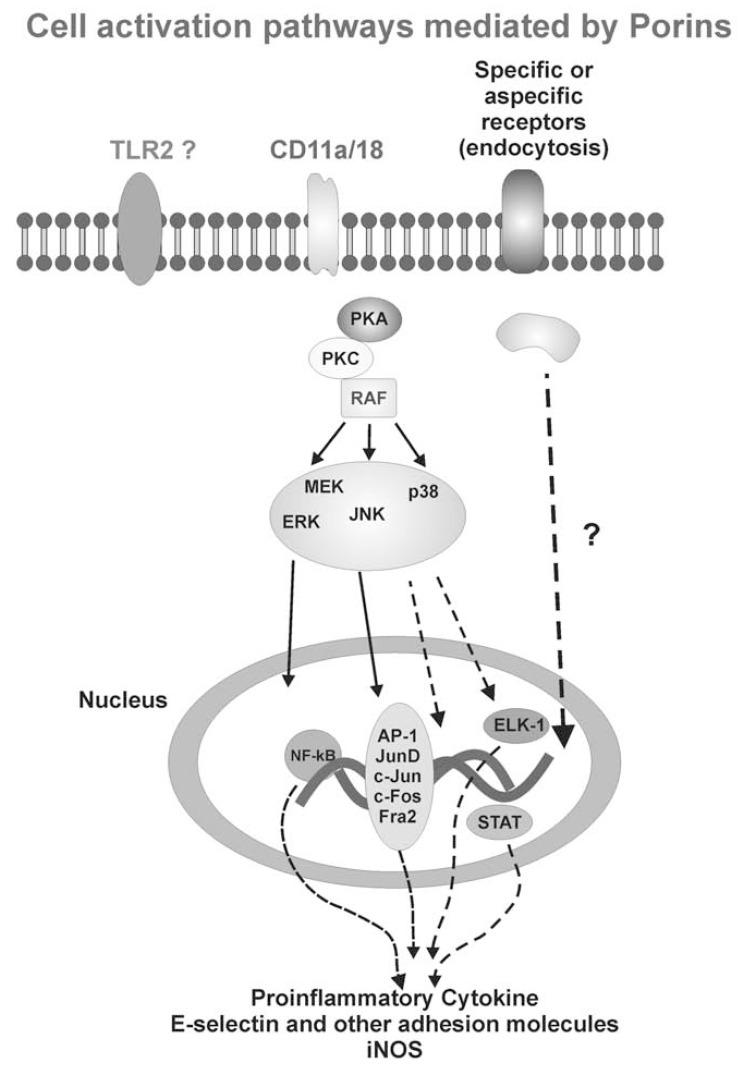
Speculative scheme of porin signal transduction pathways. Putative porin-specific receptors are shown to be transmembrane. The solid arrows indicate the known association between superficial porin receptors and activation of several transcription factors; dotted arrows indicate hypothetical events.

A speculative scheme of signal transduction pathways involved in porin-mediated responses is depicted in (Fig. **5**). Accumulating evidence has suggested that the regulation of transcriptional factors and the subunit composition by porin stimulation may affect the adaptive immune mechanism to modulate the production of biologically active proteins or peptides. The engagement of multiple pathways during signal transmission makes the possible use of molecular inhibitors as therapeutic agents very difficult; although recent findings show that peptides complementary to loop regions have a certain ability to block the activity of the porin [86].

Activation of the coagulation and fibrinolytic systems is an important manifestation of the systemic inflammatory response of the host to infection. The *in vivo* effect of a synthetic peptide corresponding to loop L7 from *Haemophilus influenzae* type b (Hib) porin was compared with the effect of the entire protein to evaluate its role on the coagulative/fibrinolytic cascade and the circulating markers of endothelial injury [87]. Plasma was obtained from rats injected intravenously with the peptide and tested for fragment 1+2 (F1+2), tissue-type plasminogen activator (tPA), plasminogen activator inhibitor type I (PAI-1) antigen, von Willebrand factor (vWF) and soluble E-selectin (sE-selectin). The coagulative/fibrinolytic cascade was impaired as determined by the increased level of PAI-1. Concomitantly, E-selectin, a marker of endothelial injury, was also significantly elevated. In addition either loop L7 or Hib porin injection induced hyperglycaemia and inflammatory cytokine production. The data were correlated with hemodynamic functions (significant reduction of blood pressure and increase of heart rate) indicate that loop L7 plays an essential role in the pathophysiologic events observed during gram-negative infections. 

## PORINS INFLUENCE ON THE EMERGENCE OF ANTIBIOTIC RESISTANT STRAINS OF PATHOGENIC BACTERIA

Multidrug resistance (MDR) is frequently reported in clinical Gram-negative bacteria. This problem poses severe limits to therapeutic options available to clinicians and is a major cause of mortality when acquired as a nosocomial infection [88,89]. MDR is prevalent in key Gram-negative clinical pathogens, such as *Escherichia coli, Salmonella spp., Klebsiella spp., Enterobacter spp., Campylobacter spp., Acinetobacter spp. *and *Pseudomonas spp*. Three major bacterial strategies have emerged for the development of drug resistance: the membrane barrier limits the intracellular access of an antibiotic; the enzymatic barrier produces detoxifying enzymes that degrade or modify the antibiotic; and the target protection barrier impairs target recognition and thus antimicrobial activity [90]. These mechanisms can act simultaneously in clinical isolates, giving rise to a high level of resistance in a single species. The OM of Gram-negative bacteria behaves as a highly selective barrier mainly through the combined effect of a hydrophobic lipid bilayer together with pore-forming proteins of specific size-exclusion properties. The permeability properties of this barrier, therefore, have a major impact on the susceptibility of the microorganism to antibiotics, which are essentially intracellularly targeted. Small hydrophilic drugs, such as β-lactams, as well as tetracycline, chloramphenicol and fluoroquinolones, use the pore-forming porins to gain access to the cell interior, while macrolides and other hydrophobic drugs diffuse across the lipid bilayer. The emergence of drug-resistant strains in a large number of bacterial species due to modifications in the lipid or protein composition of the OM highlights the importance of the OM barrier in antibiotic sensitivity. Resistance generally emerges when a decrease in the rate of entry of these compounds is generated. There are several reports where antibiotic resistance is acquired as a consequence of loss or functional impairment of porins in a large number of organisms, such as *Escherichia coli, Pseudomonas aeruginosa, Neisseria gonorrhoeae, Enterobacter aerogenes *and *Klebsiella pneumonia *[1,91-93]. There are two major porin-based mechanisms for antibiotic resistance that have been reported in clinical isolates: 1) alterations of outer membrane profiles, including either loss/severe reduction of porins or replacement of one or two major porins by another; 2) altered function due to specific mutations reducing permeability. Antibiotic-driven tetracylcine resistance can occur by exposing sensitive *Escherichia coli* cells to increasing concentrations of the antibiotic, leading to a chromosome-mediated multiple antibiotic resistance (Mar phenotype). Multiple proteins including porins and drug efflux pumps are involved, through upregulation of marA which ends up in inhibition of OmpF RNA translation [94]. Decreased OmpF levels can also derive from periplasmic accumulation of other OM proteins, such as TolC and OmpX, which might compete with the same substrate for assembly of porins in the OM [95]. The substitution of a general-diffusion porins normally present on the bacterial surface with a narrower porin is a further mechanism for acquiring antibiotic resistance. This is the case of substitution of OmpK35 and OmpK36 from K. pneumoniae with OmpK37, known to form smaller pores [96]. Reduced permeation rate as a consequence of altered function of porin represents yet another strategy found in antibiotic resistant bacteria. Several mutations in the L3 loop have been described that induce a constriction of the pore, for example, a clinical isolate of *Enterobacter aerogenes* with a glycine to aspartate substitution [97]. This mutation may cause a modification of the loop with a reduction of the pore lumen [98] and consequently a consistent decrease in porin conductance and reduction in cephalosporin sensitivity. Similar alterations have been demonstrated in the amino acid composition of the *Neisseria gonorrhoeae* porin Por [99]. Resistance to carbapenems in *P. aeruginosa* has been described in mutants lacking the porin-specific OprD and in mutants with deletions in the L2 loop of OprD [100]. These examples illustrate the role of porin channels in the mechanisms of antibiotic resistance emergence and provide molecular insights which could enable identification of new strategies to design more effective antimicrobials.

## CONCLUSION

Bacteria have developed sophisticated virulence factors such as the OM to mount their attack against the host. One of the most fascinating aspects of the OM of Gram-negative bacteria is its ability to act as a highly selective barrier mainly through the combined effect of a hydrophobic lipid bilayer together with pore-forming proteins of specific size-exclusion properties. Porins from several Gram-negative bacteria play a fundamental role in the host-pathogen interaction, eliciting diverse biological proinflammatory activities and immune responses. In fact, porins present an intrinsic biological activity when interacting with eukaryotic cells, but also behave as antigens stimulating specific immune responses. While the structure determination of β-barrel membrane proteins has benefited from both an increased interest in the field and by the advancement of new crystallization and sample preparation techniques, the field remains an exciting challenge for future research efforts. The structures of many unidentified bacterial OM proteins with unknown functions are still unsolved, and the mechanism by which β-barrels are properly folded and inserted into the OM is still poorly understood. Mechanisms affecting the barrier properties of the OM through the modification of expression and/or function of the porin channels may have an huge impact on the sensitivity of Gram-negative bacteria to antibiotics, therefore a better understanding at the molecular level of both the structure and the function of bacterial porins will allow improvements of the current drug therapies or the design of new types of antibiotics that target these surface exposed structures.
